# SOCIAL DETERMINANTS OF HEALTH AND THE RISK FOR DEMENTIA

**DOI:** 10.1093/geroni/igad104.0272

**Published:** 2023-12-21

**Authors:** Matthew Baumgart, Kischa Hampton, Chelsea Kline

**Affiliations:** Alzheimer’s Association, New York, New York, United States; Alzheimer’s Association, Chicago, Illinois, United States; Alzheimer’s Association, Chicago, Illinois, United States

## Abstract

Social determinants of health (SDOH) play a role in the risk for dementia in two ways. First, certain SDOH may be direct risk factors for dementia. Second, SDOH contribute to the prevalence of many modifiable risk factors that in turn may elevate dementia risk – and act as barriers to addressing individually modifiable risk factors for dementia. To better understand how SDOH affect the risk for dementia and the role public health has in addressing dementia-related SDOH, the Public Health Center of Excellence on Dementia Risk Reduction (PHCOE-RR), in partnership with Wake Forest School of Medicine, recently synthesized the current state of the evidence. Following that synthesis, the PHCOE-RR conducted (1) a workshop where researchers and public health officials reviewed the science, and (2) a Public Health Roundtable where the public health community discussed the role of public health agencies in addressing dementia-related SDOH. This presentation will review the results of the evidence synthesis and discuss the work of the PHCOE-RR in helping state, local, and tribal public health agencies take action in their communities.

